# Information extraction from German radiological reports for general clinical text and language understanding

**DOI:** 10.1038/s41598-023-29323-3

**Published:** 2023-02-09

**Authors:** Michael Jantscher, Felix Gunzer, Roman Kern, Eva Hassler, Sebastian Tschauner, Gernot Reishofer

**Affiliations:** 1 Know-Center, 8010 Graz, Austria; 2 Division of Neuroradiology, Vascular and Interventional Radiology, Department of Radiology, Medical University Graz, 8036 Graz, Austria; 3 Division of Pediatric Radiology, Department of Radiology, Medical University Graz, 8036 Graz, Austria; 4 Department of Radiology, Medical University Graz, 8036 Graz, Austria; 5 BioTechMed-Graz, 8010 Graz, Austria

**Keywords:** Computer science, Data mining, Machine learning, Medical imaging

## Abstract

Recent advances in deep learning and natural language processing (NLP) have opened many new opportunities for automatic text understanding and text processing in the medical field. This is of great benefit as many clinical downstream tasks rely on information from unstructured clinical documents. However, for low-resource languages like German, the use of modern text processing applications that require a large amount of training data proves to be difficult, as only few data sets are available mainly due to legal restrictions. In this study, we present an information extraction framework that was initially pre-trained on real-world computed tomographic (CT) reports of head examinations, followed by domain adaptive fine-tuning on reports from different imaging examinations. We show that in the pre-training phase, the semantic and contextual meaning of one clinical reporting domain can be captured and effectively transferred to foreign clinical imaging examinations. Moreover, we introduce an active learning approach with an intrinsic strategic sampling method to generate highly informative training data with low human annotation cost. We see that the model performance can be significantly improved by an appropriate selection of the data to be annotated, without the need to train the model on a specific downstream task. With a general annotation scheme that can be used not only in the radiology field but also in a broader clinical setting, we contribute to a more consistent labeling and annotation process that also facilitates the verification and evaluation of language models in the German clinical setting.

## Introduction

With the wide application of artificial intelligence in medicine, there is an increasing need for the analysis of medical texts^1–4^. Structured text data form the basis for many information retrieval use cases^5–11^ like Clinical Decision Support (CDS), diagnostic surveillance, cohort building for epidemiological studies, or query-based case retrieval. However, extracting structured and normalized information from clinical documents is a challenging task due to the lack of consistent language and standardized reports. Clinical documents, especially radiological reports, differ greatly in writing style from general medical documents such as scientific papers and articles. Due to time constraints, these documents/reports written by clinical staff are brief and concise and cover only important medical information (telegram style) with a subordinate focus on grammatical correctness. This leads to a pronounced divergence in semantics as well as syntax to common language.

[Editor1.1] Recently, deep language models, like bidirectional encoder representations from transformers (BERT)^12^, have shown an impressive performance boost for various NLP downstream tasks in the German clinical domain, such as (i) information extraction from radiological reports^13–16^, (ii) free-text report classification^17,18^ and (iii) oncology report summarization^19^. However, training large language models requires a significant amount of well-annotated training and testing data. Although hospitals already collect a vast amount of valuable digital free-text data (discharge reports, radiological reports, etc.) every day, they cannot be made accessible for external research due to privacy concerns and local legal restrictions. The situation is even worse for low-resource languages like German and hampers the development of modern healthcare applications in this field.

There are several initiatives for information extraction in German clinical documents derived from different medical fields. Roller et al.^13^ introduced a workbench for information extraction on German nephrology reports. Others focus on data from echocardiography reports ^14^, mental health records^15^ or self-generate synthetic clinical data^16^. However, these studies focus on a specific medical dataset, and may not allow validation of their approach in a broader clinical setting. In addition, there is a lack of a commonly agreed annotation, which makes comparison and validation with others difficult. Biased language models are one of the main resulting drawbacks^20,21^.

**Figure 1:**
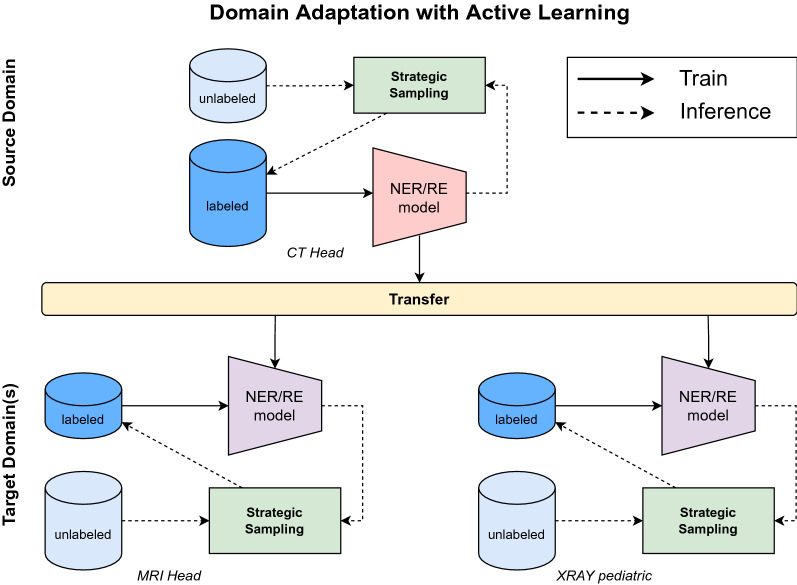
Domain adaptation with active learning leveraging a strategic sampling strategy.

In this work, we contribute to an emerging field of research that emphasizes the role of information extraction techniques in the medical domain for low-resource languages like German. Our goal is to provide a universal German radiological language model that can be transferred to other clinical fields with minor adaptations (see Fig. 1). This allows other research teams to fine-tune the model on local datasets for specific clinical use cases without the need for expensive computing and human resources. These clinical applications cover (i) predictive clinical tasks, (ii) the generation of research cohorts, or (iii) the generation of image labels for upcoming AI-based medical imaging tasks.

In this paper, we pay attention to radiological reports due to their lower syntactic complexity compared to other clinical documents. Unlike clinical documents such as discharge letters or surgery reports, imaging reports focus on specific anatomical regions and consequently specific pathologies and observations. This reduces the amount of potential clinical and medical information. Therefore, radiological reports are a good starting point for the development of a German clinical language model. In order to train this language model, we introduce active learning along with a strategic sampling method to generate highly informative labeled training data (Fig. 1). This process of data labeling is particularly challenging in the clinical setting, as the complexity of clinical texts requires the involvement of medical experts in the labeling process. As a final step, we present a general annotation scheme for named entity recognition (NER) and relation extraction (RE) that not only considers the radiological domain but a broader clinical setting.

We make the following contributions to the interdisciplinary field of natural language processing and clinical research:

- We provide a transformer-based language model that has been trained on German radiological reports for the tasks of (i) NER and (ii) RE. We emphasize, that our language model provides a significant boost in performance for medical predictive studies. To the best of our knowledge, this work is the first German radiological language model which can be used as a starting point for many clinical downstream tasks.
- By implementing active learning along with strategic sampling, we present an efficient method to generate consistently labeled training data with little annotation effort. We show that this method, in combination with a pre-trained language model, has great potential for general knowledge acquisition across different imaging domains.
- We present a general annotation scheme that includes supervision from radiologists, incorporation of medical ontologies (RadLex, MesH) and previous work^22,23^ that can be used not only in the radiological field but also in a broader clinical setting. We therefore contribute to a more consistent labeling and annotation process, which is also beneficial in verifying and comparing modeling approaches in the German clinical domain.

## Results

### Experimental setup

For this study, we obtained radiological reports from three different imaging modalities from our institution. Table 1 gives an overview of the different training and test sets. Sentence count states the number of unique sentences per dataset. Based on the schemata from Tables 4, 5, clinical entities (Named Entity) and relationships (Relationship$^+$ and Relationship$^-$) between those entities were extracted from the reports, using an active learning approach. Relationship$^+$ refers to actual relations of entities while entities that have no relationship with each other within a sentence (Relationship$^-$) are automatically labeled as $no\ relation$.

**Table 1:** Dataset overview.

Dataset	Sentence count	Entity count		
		Named entity	Relationship$^+$	Relationship$^-$
Training				
CT Head (CTH)	1398	6174	16,780	28,916
MRI Head (MRIH)	512	2878	3156	20,732
Xray pediatric (XPED)	512	4485	4138	34,588
Test				
CT Head (CTH)	1074	3992	4381	25,004
MRI Head (MRIH)	423	1852	1901	9230
Xray pediatric (XPED)	213	1012	701	6578

Number of unique records per dataset as well as number of extracted (clinical) named entities and relationships.

A pre-trained BERT language model is fine-tuned for (i) NER and (ii) RE. Following the masked language modeling method, 15% of the words in the CT Head (CTH) dataset were randomly masked and used for self-supervised pre-training. The pre-trained model serves as a basis for further fine-tuning in the following experiments. A more detailed description of the active learning procedure can be found in the “Material and Methods” section.

### Results for CT Head reports (source domain)

**Table 2:** Comparison of sentences with different perplexity scores (pps) and lengths.

Strategy	Sentence
Short sentence with low pps	Keine rezente Blutung *(No recent bleeding)*
Long sentence with low pps	Geringgradige randständige Schleimhautschwellung im Sinus frontalis, sowie im Sinus maxillaris rechtsseitig, im Übrigen regelrechte Belüftung der Nasennebenhöhlen und des Mastoidzellsystems beidseits *(Minor borderline swelling of the mucous membrane in the frontal sinus and* *in the maxillary sinus on the right side, otherwise normal ventilation of the* *paranasal sinuses and the mastoid cell system on both sides)*
Short sentence with high pps	Mikrophtalmie rechts mit verkalktem Bulbus oculi *(Microphtalmia on the right with calcified bulbus oculi)*
Long sentence with high pps	Im Wesentlichen unveränderte postoperative Verhältnisse in der hinteren Schädelgrube bei Zustand nach Teilentfernung eines Vestibularisschwannoms am Kleinhirnbrückenwinkel rechts (*Essentially unchanged postoperative conditions in the posterior fossa* *after partial removal of a vestibular schwannoma at the right* *cerebellopontine angle*)

**Figure 2:**
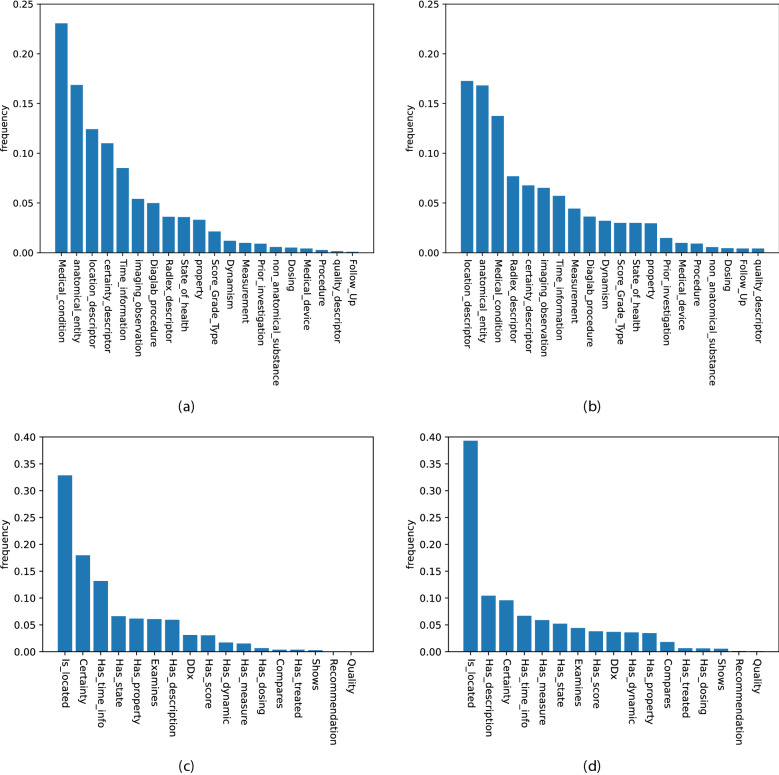
Distribution of named entity classes (**a**) in the random sampled and (**b**) in the strategic sampled training set as well as the relationship classes (**c**) in the random sampled and (**d**) in the strategic sampled training set (CTH training data set).

In this first experiment, the language model is fine-tuned on labeled data derived from the CTH dataset. Initially, 268 randomly selected sentences were annotated by two clinical experts. During the first seven learning cycles, 100 additional randomly selected samples were chosen from the pool of unlabeled sentences per iteration and proposed to the clinical experts for annotation.[Rev1.1] During the random sampling iterations, a subset of 20% of the sentences per iteration was annotated by both clinical experts. In addition, 10% of all sentences were annotated by both clinical experts during the strategic sampling iterations. Based on these expert reviews, an inter-annotator agreement was estimated separately for both the random sampling phase and the strategic sampling phase. The Cohen’s kappa values of 0.74 (random sampling) and 0.83 (strategic sampling) show a substantial as well as a near perfect agreement between the annotators. Inspired by Ramponi and Plank^24^ as well as Salhofer et al.^25^ we intervene at iteration 8 with a strategic sampling approach that favors longer sentences/samples with a higher perplexity score (Eq. 1). In order to ignore outliers but still select highly informative training samples, we select samples (sentences) in the upper 75% to 90% percentile of the perplexity scores and sentence lengths. Examples of sentences with different lengths and perplexity scores are stated in Table 2. It can be observed that sentences containing medical entities such as diseases that are more common in reports (e.g., regular sinus ventilation) lead to low perplexity values. Sentences containing rare diseases (or medical conditions), like “calcified bulbus oculi”, are much more surprising to the model and lead to higher perplexity scores. We conclude that drawing longer sentences with a higher perplexity score leads to samples with a higher number of named entities and, implicitly, more relationships compared to the random sampling strategy. The values in Table 3 support this conclusion. The second advantage of this strategic sampling approach is that entity and relationship classes are more uniformly distributed in the training set. Figure 2 shows that the distributions are much more skewed toward the minor classes in the strategic sampling method.

**Table 3:** Overview CT Head training sets per active learning iteration.

Sampling strategy	Number of sentences	Avg. sentence length	Avg. perplexity score	Avg. number of entities per sentence		
				Named entity	Relationship$^+$	Relationship$^-$
Random sampling	[Rev1.2] 268	45	1.25	2.60	13.3	9.40
	100	44	1.27	3.68	11.56	10.92
	100	38	1.12	3.64	6.84	7.64
	100	41	1.22	3.75	8.16	12.82
	100	39	1.25	3.20	7.16	7.64
	100	37	1.26	3.20	7.72	8.70
	100	47	1.19	3.19	4.08	4.42
	100	44	1.20	3.67	2.36	2.72
Strategic sampling	100	90	3.41	10.70	27.14	90.30
	100	77	2.72	8.50	17.00	39.34
	100	62	2.21	4.70	14.24	20.44
	100	56	1.96	4.22	15.04	19.50

By starting with an initial amount 268 labeled sentences, in each iteration additional sentences have been annotated and added to the training set. Using strategic sampling increases the number of occurrences of named entities and relationships within sentences compared to random sampling.

**Figure 3:**
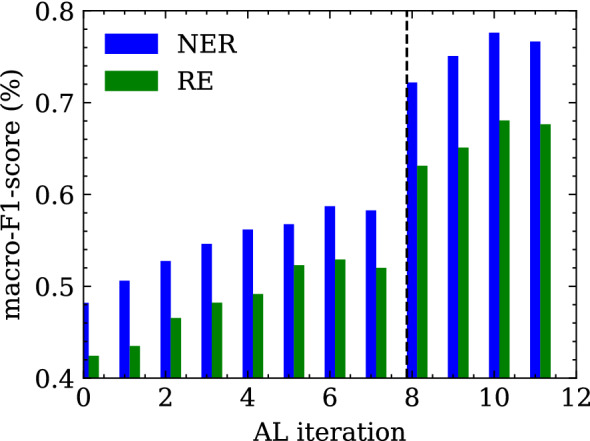
Macro F1-score over the different active learning cycle iterations for both tasks (i) named entity recognition and (ii) relation extraction evaluated on the CTH test set. The vertical line at iteration 8 marks the change from random sampling to a strategic sampling approach.

**Figure 4:**
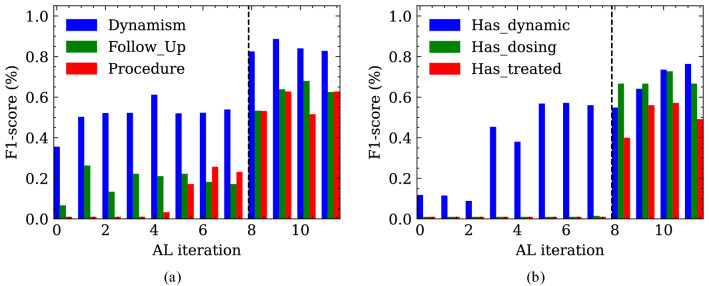
Improvements for (**a**) named entity classification and (**b**) relationship classification on the CT Head test data set after applying strategic sampling for active learning. The vertical line at iteration 8 marks the change from random sampling to a strategic sampling approach.

As the vast majority of relationships correspond to the None relations (Relationship$^-$), the positive relations (Relationship$^+$) were oversampled four times to avoid strong class imbalance during training. Since the model is to be evaluated based on the actual distribution of relationship classes, no oversampling is performed for the test set. To confirm the performance of the language model in terms of NER and RE within (i) the same imaging modality and (ii) the same anatomical region, the evaluation is based on the CTH test set. Since we assume that each entity and relationship class is equally important, the macro F1-score is used as an evaluation measurement. In Fig. 3, the main results are reported. The score slightly increases during the iterations where random sampling is applied. At iteration 8, when we intervene with the strategic sampling approach, the score increases dramatically until it reaches a saturation range. There are two reasons for this increase in performance: (i) the training set contains many more sentences with a larger number of clinical entities and relationships, and (ii) the distribution of the different classes in the training set when strategic sampling is applied is more uniform than in the training set when random sampling is applied (Fig. 2). Figure 4 visualizes the entity classes, which benefit the most from the intervention with strategic sampling. Again, the saturation range is reached quite fast after the intervention.

### Transfer to another clinical domain (domain adaptation)

Medical entities as well as the writing style may differ between clinical reports due to (i) the type of clinical examination (ii) the anatomical region and (iii) the individual preferences of the medical experts. Although a pre-trained language model is biased toward a specific clinical domain, we want to emphasize that it is still beneficial to fine-tune it for a foreign clinical domain. This is especially true when only a small training dataset is available.

Current research in the field of domain adaptation can be categorized into model-centric, data-centric and hybrid as described in the work of Ramponi and Plank^24^. Since we already use active learning and strategic sampling for model training in the first experiment, and large unlabeled datasets are available in the foreign domains, we continue with this data-centric data selection approach for domain adaptation.

#### Results for MRI Head reports

**Figure 5:**
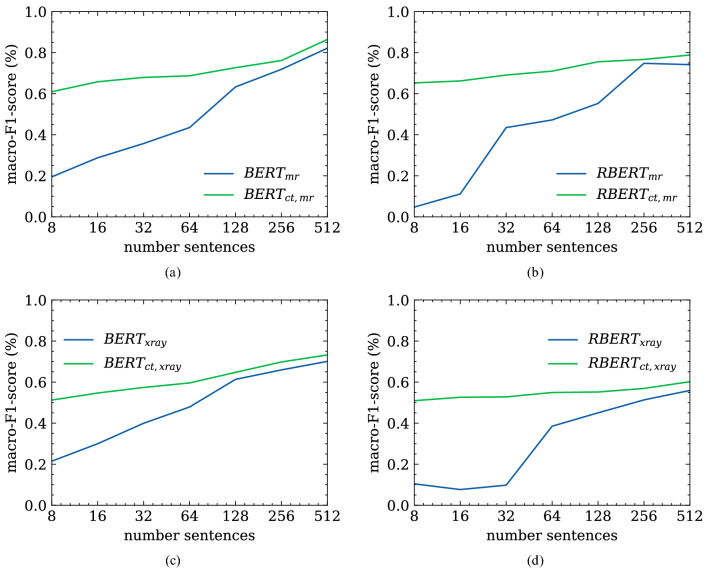
Performance of (**a**) NER and (**b**) RE models on the MRIH dataset as well as the performance of the (**c**) NER and (**d**) RE models on the XPED set. Domain adapted models, prior trained on a dataset from a foreign clinical domain, result in improved performance compared to models trained on a small dataset from a single domain.

In a second experiment, we validate a fine-tuned NER and RE model adapted to a foreign imaging domain (Magnetic Resonance Imaging). Hence, we compare the models that were (a) previously trained on the CTH dataset and now fine-tuned on the MRI Head (MRIH) training set ($BERT_{ct,mr}$ and $RBERT_{ct,mr}$) with those that were (b) trained on the MRIH dataset only ($BERT_{mr}$ and $RBERT_{mr}$). After several active learning iterations with strategic sampling on the MRIH training set, we see that the $BERT_{ct,mr}$ model outperforms the $BERT_{mr}$ model (Fig. 5). The same applies to the RE task. This is especially true for the very early stages where the amount of training data is quite small.

#### Results for Xray pediatric reports

In the third experiment, we do not only switch to another imaging modality but also to a different anatomical region where the examination is performed. Like in the experiment before we compare the models previously trained on the CTH reports and fine-tuned on the Xray pediatric (XPED) dataset and the models trained on the XPED reports set only. It is shown that although the performance of the $BERT_{xray}$ and $RBERT_{xray}$ increases with growing training set, the multi-domain models $BERT_{ct, xray}$ and $RBERT_{ct, xray}$ are dominating (Fig. 5).

## Discussion

In this work, we present a pre-trained and fined-tuned German language model (BERT and RBERT) for the task of information extraction (NER and RE) in the radiological domain. This model was trained on clinical reports derived from three different types of imaging examinations (CT, MRI, radiograph). In order to standardize and support further research in this area, we introduced a general annotation scheme that was developed together with clinical experts based on previous research and common medical ontologies. Recent work from Irvin et al.^22^ and Jain et al.^23^ introduced annotation schemata for the classification of radiology reports. They mainly focus on the classification of reports based on pathologies and/or anatomical regions which can be used as image labels for further research in the field of AI-based medical imaging. In contrast, our annotation scheme extends their work in a way that allows for more general clinical knowledge acquisition from documents since radiology reports contain not only pathologies and anatomical entities, but more detailed clinical information. For example, the medical reason why a patient is requested to undergo an imaging examination or the possible diagnosis given by the radiologist. Considering this additional clinical information, a model trained on our schema enables a broader range of clinical research tasks. For example, not only (i) classifications tasks (e.g. aneurysm: yes/no) but also (ii) query-based case retrieval for cohort building studies (e.g. from all medical records, return patients with one or more aneurysms >7mm of the A. cerebri media (ACM)) or (iii) diagnostic surveillance (e.g has the aneurysm increased/decreased in size or remained the same compared to the previous examination?). Various clinical downstream tasks can be dynamically defined and formulated in a post-processing step based on the annotation schema without the need to train separate models. We are aware that not all classes are required for every clinical downstream task. Therefore, decisions about relevant entity and relationship classes must be made in collaboration between medical experts and data scientists prior to annotation. These decisions depend heavily on the (i) medical data and (ii) the clinical use case. Active learning in combination with strategic sampling based on intrinsic measurements allow us to efficiently generate highly informative labeled training data at low cost. A major advantage of intrinsic measurements over extrinsic measurements is that they do not rely on language models trained on a specific downstream task (like NER or RE) and therefore do not require labeled samples. Instead, a self-supervised, pre-trained model forms the basis for these sampling strategies without the need for expensive, human-annotated data. However, the most popular strategies like uncertainty sampling^26^ or margin-based sampling techniques^27,28^ rely on extrinsic measurements. These sampling techniques have a major drawback when the annotation scheme (entities and relationship classes) changes, for example, due to domain adaptation. In this case, the sampling strategy must be manually adapted to the new entity and relationship classes. Our intrinsic strategic sampling technique removes this drawback and provides a more general approach based only on raw report texts. Moreover, we conducted experiments based on two intrinsic sampling strategies (i) random sampling and (ii) strategic sampling based on the length and the perplexity score of a sentence. In our study, we found that strategic sampling outperformed our baseline random sampling method. Strategic sampling favors longer sentences with a high perplexity score. Since annotation is mostly performed by medical experts due to the complexity of clinical documents, our sampling approach in combination with active learning provides a highly efficient and time-saving framework for creating training datasets. For the task of information extraction from clinical records (i) the German-MedBERT^29^ and (ii) R-BERT^30^ models were used as a starting point for named entity recognition and relation extraction, respectively. To gain a linguistic understanding of clinical documents and to account for their specific writing style, the models were pre-trained using masked language modelling on a German clinical corpus. Multiple studies have demonstrated the advantage of adaptive pre-training under both high- and low-resource settings^31,32^. In the first experiment, we fine-tuned and evaluated the NER and RE models on labeled samples from the CTH dataset. We found that by applying active learning with strategic sampling, our method achieved improved results for the NER and the RE task, respectively, considering the amount of available labeled training data. Underrepresented classes in particular benefit from this training setup, as the representation in the training set is much more balanced (Fig. 2) which further improves the overall performance of the model, as visualized in Fig. 3.

As we observed in further experiments, the performance of the language models highly depends on the training set size. Since many research institutions lack sufficient clinical data to train deep-learning language models from scratch, transferring knowledge from trained models from a foreign clinical domain mitigates the problem of poor model performance and overfitting. In terms of domain adaptation in the clinical sense, we distinguish two different scenarios in these experiments: (i) change in the type of radiological examination (from CT scans of the head to MRI scans of the head) and (ii) change in the type of radiological examination and the anatomical region for which the examination is performed (from head-specific examinations to whole-body examinations). For both scenarios, the pre-trained model, fine-tuned on the respective foreign dataset, outperforms the language model trained from scratch. Especially for smaller foreign datasets, differences in performance become even more apparent (Fig. 5).

A closer look shows that the overall performance of the models trained on the MRIH dataset (Fig. 5a,b) with 86% for $Bert_{ct,mr}$ and 80% for $RBERT_{ct,mr}$) is much higher than those of the models trained on the XPED dataset (Fig. 5c,d) with 74% $Bert_{ct,xray}$ and 60% $RBERT_{ct,xray}$) considering the same amount of training data. This is due to the fact that the CTH and MRIH datasets share similar entity distributions. Although the imaging modalities are different, essential parts such as anatomical entities (cranium, ventricle, basal ganglia), medical conditions (acute sinusitis, territorial ischemia, Basal ganglia calcification) as well as the writing style itself are similar according to the experts. The main reasons for this are that (i) CT and MRI scans of the head are performed by the same radiological department (neuroradiology) so that the same anatomic region and anatomic structures are examined, and (ii) MRIs are often performed as a follow-up to a CT scan, for example in an emergency case. Therefore, we observe dependencies/overlaps between reports of these imaging modalities, which in turn facilitate the domain adaption process.

In contrast, the CT head dataset and the pediatric radiograph dataset differ both in writing style and in the medical information included. This is because (i) the imaging study focuses on different anatomical regions and pathologies (ii) the clinical questions differ from each other and ultimately (ii) due to the different imaging modalities. The work of Casey, Alene et al.^33^ and Zech et al.^34^ support our findings in that the highly specialized nature of each imaging modality lends itself to the creation of sublanguages. These different writing styles across imaging modalities pose an additional challenge for a language model. Moreover, pediatric radiographs have a different prevalence of pathological findings than CT and MRI examinations due to different patient populations. In our study, CT and MRI examinations were performed on elderly patients who usually have a longer medical history, whereas children who underwent a radiographic examination usually suffered from acute medical conditions. All these aspects have a major impact on the task of information extraction and therefore must be considered when training a language model from scratch or adapting it to a foreign clinical domain.

Our work has certain limitations. First, this is a single-center study and other open data sources in this area are scarcely available to evaluate our work. The performance of the information extraction approach may vary across clinical sites because (i) different specifications for the format of clinical reports (structured vs unstructured) result in different writing styles and (ii) disease prevalence varies by demographic factors. In addition, we currently cover a specific area in the clinical field where reports are used as a communication tool. Therefore, we encourage further research to evaluate our open language models in a different clinical domain and use them as a starting point for further research. The annotation and training strategies described in this paper should facilitate the whole process of training data generation and lead to more unified research in the field of German clinical information extraction.

## Conclusion

[Rev1.6] In this study, we demonstrate the feasibility of clinical language understanding in low-resource languages such as German. Combined with an efficient training and labeling strategy and a standardized annotation scheme, this work forms the basis for various clinical prediction tasks and encourages others to contribute to more consistent work in this field. Especially clinical studies with a small data set benefit from pre-trained German language models. [Rev2.2] We have shown that strategic sampling has great potential for effective selection of training data and minimizes the cost of human annotation. Validation as well as comparison with other sampling strategies need to be performed in future experiments on heterogeneous clinical datasets. [Rev1.4], [Rev2.3] In future studies, the language models will be extended and validated on datasets from other imaging disciplines derived from foreign clinical institutions as well as documents from other clinical disciplines such as discharge letters. Good starting points for this are (i) open datasets from the MIMIC-IV database or (ii) n2c2-NLP research datasets that could be translated into German or used directly for multilingual language modeling. In addition, we plan to collaborate with other German-speaking clinical institutions to expand the scope with real-world clinical data.

## Material and methods

### Ethics declarations

All patients gave written informed consent to participate in this study. The study was approved by the ethics committee of the medical university of Graz according to the guidelines of the declaration of Helsinki.

### Study cohort and preprocessing

In this study, radiological reports of CTs of the head were retrieved between 2015–2021 from the radiological department. After data preprocessing and cleaning, 88.467 distinct reports remained for further analysis.

In addition, 100 reports of MRI examinations of the head focusing on cerebral aneurysms were collected and used for domain adaptive training and validation respectively.

Further, data was derived from the department of pediatric radiology. These 74.183 reports are based on radiographs of different body parts of children.

The preprocessing steps of all three datasets cover (i) text extraction from the “findings”, “impression”, “history” and “comparison” sections of a radiological report and (ii) sentence splitting, using the SoMaJo library^35^. [Rev2.4] Tokenization of words and subwords is performed directly by the BERT language model during training and inference using the concept of WordPiece tokenization. These pre-processed reports are converted to the Brat-standoff format, which serves as the basis for the human annotation process. All datasets were acquired during clinical routine and retrospectively analyzed.

**Figure 6:**
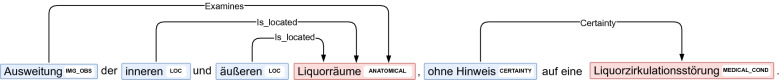
Example of an annotated sentence of a radiological report. English translation: expansion of the internal and external CSF spaces, without evidence of a CSF outflow disorder.

### Annotation guidelines and corpus generation

The annotation guidelines for entities and relationships were carried out by radiologists from our clinical department. Based on medical ontologies (UMLS, RadLex, MeSH) and prior work^13,36,37^, the annotation scheme covers relevant clinical information with a focus on radiological reports. However, we emphasize to keep annotation classes as general as possible so that the task of information extraction can be easily transferred to other clinical domains with only minor adjustments. Tables 4, 5 describe entity and relationship classes, respectively. The annotation task was performed using the BRAT annotation tool^38^. New annotated samples were iteratively added to the training set depending on the active learning strategy. Each document in the training set was examined by two radiologists.

### Named entity recognition

For the task of clinical named entity recognition, we rely on a German pre-trained clinical language model^29^. We continue self-supervised pre-training using masked language modeling (MLM) with the whole set of radiological CT reports of the brain. Self-supervised pre-training uses rich unlabeled text data to train the transformer network and obtain a contextual word vector representation. This model builds the backbone for the downstream tasks (i) named entity recognition as well as (ii) relation extraction.

#### Fine-tuning for NER

To ensure that the language model knows that an entity can be a single word or a group of words, we need to provide information about the beginning and ending of an entity in our training data. In this work, this is realized via the IOB (Inside Outside Beginning) tagging schema. Further, we replace the pre-training output layer with a linear layer for the task of entity classification. Each entity class, stated in Table 4, is defined in two ways, either with the “B-<class name>” or “I-<class name>”. For example, the phrase “ohne Hinweis” (no indication) in Fig. 6 would be split into “B-Certainty” and “I-Certainty” following the IOB labeling schema. Further, all unlabeled tokens like “der”, “und”, auf”, “ein” will be labeled with the “O” tag, representing the class *no entity*.

### Relation extraction

#### Sample generation

In this section, we describe the task of within-sentence relation sample generation and classification. Given a radiological report *D* that contains a set of *n* sentences $\{s_i\}_{i=1}^{n}$. Each sentence $s_i$ holds a set of $k_i$ named entities $\{e_j\}_{j=1}^{k_i}$, extracted prior by the the entity recognition model. The relation extraction task infers the relation between entity pairs $(e_n, e_m)$, where the subscripts *n* and *m* relates to distinct entities within a sentence. The training set of relations refers to the joint set of annotated relations $R^+$ and the set of *no relation* $R^-$: $R_{train} = R^+\cup R^-$. By definition, entities which have no relation to each other within a sentence are automatically labeled as *no relation*. Since we do not consider the direction of the relation, all possible combinations (relations) between two distinct entities within a sentence can be expressed by: $$\left( {\begin{array}{c}k_i\\ 2\end{array}}\right)$$. According to the sentence in Fig. 6, 6 entities can be extracted. Thus, 15 relations are possible, four of which are positive relations. The remaining 11 relations are labeled as *no relation*.

#### Model architecture

Our supervised relation classification approach is based on an pre-trained relation extraction model called R-BERT^30^. As a starting point we use the pre-trained language model from the previous section. Compared to the standard relation classification approach, we adapted the model output so that we do not consider the direction of the relation. This means, $Is\_located(e_1, e_2)$ is the same as $Is\_located(e_2, e_1)$. Since we incorporate predefined relationship rules, as specified in Table 5, the complexity of the final model layers is reduced which is beneficial in cases of a small training set.

### Active learning cycle

Our active learning cycle can be divided into the following phases:

1. *Initial training sample generation.* At the beginning, we randomly select an initial set of of unlabeled sentences for human annotation. The initial pre-trained language model is fine tuned for NER and RE on this set.
2. *Querying strategy.* Additional unannotated sentences are selected based on a querying strategy. The number of sentences proposed to the human annotator for labeling/verification is a hyperparameter in our AL framework and is set by default to 100 samples per iteration. In the experiments, the selected sentences were automatically labeled by the language model prior the human annotation/review phase. This reduces the cost of human annotation because the annotation of a sentence does not have to be started from scratch, but instead the already annotated labels are corrected. These automatically inferred labels are further defined as soft labels.
3. *Training NER and RE model.* The two models are retrained on the updated annotated training dataset.

Step 2 and 3 are repeated iteratively until a stopping criteria is met. In our work, we stop annotation when the accuracy reaches a threshold on a hold-out test set. This test set is generated from the same domain as the training dataset.

#### Random selection

We randomly select queries from the set of unlabeled data. The assumption is, that by random selection, (i) the underlying data distribution is represented in the training set and (i) the NER/RE models are provided with “average” informative samples. This query strategy can be performed independently of the classifier model and is the most favorable in terms of runtime and computational cost.

#### Pseudo-perplexity score

This sampling strategy relies on querying sentences with high pseudo-perplexitiy scores. Given a tokenized sequence $X=(x_0, x_1, \ldots , x_n)$, the pseudo-perplexity of this sequence is defined by:

1 $$\begin{aligned} PPL(X) = exp\{-\frac{1}{n}\sum _i^n log~p_\theta (x_i|X_{-i})\} \end{aligned}$$

   Where each token $X_j$ is masked out and predicted based on all previous and following tokens $X_{-i} = (x_0, x_1, \ldots , x_{i-1}, x_{i+1}, \ldots , x_n)$ The pseudo-perplexity of a masked language model is an intrinsic measure of how well the model is able to predict sentences in a corpus^39^. We claim that sentences where the model is least accurate (or most surprised) during inference have a higher contribution in the training phase. Compared to the random selection strategy, the queries rely on the performance of a pre-trained language model and are therefore more computationally intensive.

**Table 4:** Overview entity classes.

	Concept	Description	Example
Central	Medical_condition	Pathologic findings of disease value or findings of uncertain disease value	“akute sinusitis frontalis”, “Ausweitung e vacuo”, “Kalzifizierung”
	Anatomical_entity	Anatomical terms	“Gehirnschädel”, “Ventrikel”
	Imaging_observation	Objective radiological descriptions	“Ausweitung”, “Raumforderung”
	Diaglab_procedure	Imaging modalities, but also preliminary examinations, or clinical examinations outside of radiology	“CT”, “EKG”
	Non_anatomical_substance	Drugs, noxious agents, or other biologically active substances	“Kontrastmittel”, “Marcumar”, “Gadolinium”
	Procedure	Terms for interventions such as surgical procedures or radiation treatments	“Bohrlochtrepanation”
	Medical_device	Medical devices and materials	“Clips”, “Ventrikelsonde”
	Follow_Up	Recommendations or statements on follow-up investigations	“Kontrolluntersuchung empfohlen”, “Follow-up Untersuchung”
	Prior_investigation	Preliminary examination carried out	“Voruntersuchung”, “VU”, “MRT-Voruntersuchung”
Descriptors	Location_descriptor	Terms with spatial information, also anatomical-spatial words	“frontal”, “links”, “beidseits”
	Certainty_descriptor	Negations and speculations as well as information about the degree of certainty of findings or diagnoses	“kein”, “wahrscheinlich”, “deutlich”
	Quality_descriptor	Term about the assessability of imaging (usually in combination of an artifact)	“eingeschränkte Beurteilbarkeit”
	Radlex_descriptor	Terms that describe other entities in more detail and cannot be assigned to any other descriptor	“hypodens”, “inhomogen”, “klein”
Specifications	Dosing	The dosage of medical or non-medical substances and the radiation dose	“400 MG J/ML - 200 ML, 50.00 ml”
	State_of_health	Terms describing norm variants or normal findings.	“altersgerecht”, “frei”, “unauffällig”
	Measurement	Measurement of a parameter with following unit or dimensionless (no dosages)	“8 cm”, “30 min”, “Segment IV”
	Time_information	Terms with temporal information (no dynamic temporal changes)	“akut”, “rezent”, “vor 2 Tagen”
	Dynamism	Descriptions of dynamic changes	“neu”, progredient”, “vorbekannt”
	Property	Property or function terminology	“Durchmesser”, “Belüftung”
	Score_Grade_Type	Terms of classification, categorization, medical scores, graduations, variations, and typifications	“ASPECTS”, “geringgradig”, “Typ 2”

**Table 5:** Overview relationship classes.

	Relation	Description
Medical	Shows	Links a DiagLab_procedure with various findings that are displayed by the respective imaging method
	Examines	This relation refers to imaging modalities that examine specific anatomical structures
	Compares	Relates to findings from previous examinations that are compared with current findings such as imaging_observations or Medical_conditions
	DDx	Terms that represent possible explanations (differential diagnoses) for findings in a report
	Recommendation	Findings or conditions a follow-up examination is recommended for
Description	Has_state	Medical_condition or anatomical_entity whose state is further described by an imaging_observation
	Has_property	A term that gives various findings a property
	Has_dosing	Links the dosage with a respective non_anatomical_substance
	Has_time_info	Links Time_information entities with various terms
	Has_measure	Assigns text information containing measurements to different entities (i.e. Scores_Grade_Type, property, Medical_condition, ...)
	Has_dynamic	Associates terms that imply dynamic changes with findings in a report
	Has_score	This relation connects Score_Grade_Type entities with other terms they refer to
	Has_treated	Applied on procedures used as treatment for a Medical_condition
	Is_located	Links location_descriptor or anatomical_entity with findings which are localized by them
	Certainty	Associates terms giving information on how certain a specific finding is

## Data Availability

The datasets used and analysed within this study are not publicly available due to legal restrictions but are available from the corresponding author on reasonable request.

## Code availability

The source code as well as the pre-trained models will be available on github.
